# SnapKin: a snapshot deep learning ensemble for kinase-substrate prediction from phosphoproteomics data

**DOI:** 10.1093/nargab/lqad099

**Published:** 2023-11-06

**Authors:** Di Xiao, Michael Lin, Chunlei Liu, Thomas A Geddes, James G Burchfield, Benjamin L Parker, Sean J Humphrey, Pengyi Yang

**Affiliations:** Computational Systems Biology Group, Children’s Medical Research Institute, The University of Sydney, Westmead, NSW 2145, Australia; School of Mathematics and Statistics, The University of Sydney, Sydney, NSW 2006, Australia; Computational Systems Biology Group, Children’s Medical Research Institute, The University of Sydney, Westmead, NSW 2145, Australia; Computational Systems Biology Group, Children’s Medical Research Institute, The University of Sydney, Westmead, NSW 2145, Australia; Charles Perkins Centre, The University of Sydney, Sydney, NSW 2006, Australia; School of Environmental and Life Sciences, The University of Sydney, Sydney, NSW 2006, Australia; Charles Perkins Centre, The University of Sydney, Sydney, NSW 2006, Australia; School of Environmental and Life Sciences, The University of Sydney, Sydney, NSW 2006, Australia; Centre for Muscle Research, Department of Anatomy and Physiology, School of Biomedical Sciences, Melbourne, VIC 3010, Australia; Charles Perkins Centre, The University of Sydney, Sydney, NSW 2006, Australia; School of Environmental and Life Sciences, The University of Sydney, Sydney, NSW 2006, Australia; Murdoch Children’s Research Institute, The Royal Children’s Hospital, Melbourne, VIC, 3052, Australia; Computational Systems Biology Group, Children’s Medical Research Institute, The University of Sydney, Westmead, NSW 2145, Australia; School of Mathematics and Statistics, The University of Sydney, Sydney, NSW 2006, Australia; Charles Perkins Centre, The University of Sydney, Sydney, NSW 2006, Australia

## Abstract

A major challenge in mass spectrometry-based phosphoproteomics lies in identifying the substrates of kinases, as currently only a small fraction of substrates identified can be confidently linked with a known kinase. Machine learning techniques are promising approaches for leveraging large-scale phosphoproteomics data to computationally predict substrates of kinases. However, the small number of experimentally validated kinase substrates (true positive) and the high data noise in many phosphoproteomics datasets together limit their applicability and utility. Here, we aim to develop advanced kinase-substrate prediction methods to address these challenges. Using a collection of seven large phosphoproteomics datasets, and both traditional and deep learning models, we first demonstrate that a ‘pseudo-positive’ learning strategy for alleviating small sample size is effective at improving model predictive performance. We next show that a data resampling-based ensemble learning strategy is useful for improving model stability while further enhancing prediction. Lastly, we introduce an ensemble deep learning model (‘SnapKin’) by incorporating the above two learning strategies into a ‘snapshot’ ensemble learning algorithm. We propose SnapKin, an ensemble deep learning method, for predicting substrates of kinases from large-scale phosphoproteomics data. We demonstrate that SnapKin consistently outperforms existing methods in kinase-substrate prediction. SnapKin is freely available at https://github.com/PYangLab/SnapKin.

## Introduction

Protein phosphorylation, one of the most pervasive cell signalling mechanisms, regulates a broad range of fundamental processes such as cell metabolism (1), differentiation (2) and the cell cycle (3), and its dysregulation leads to various diseases, including cancers (4). Central to phosphorylation are the kinases that phosphorylate specific sites on their target substrate proteins. Together, kinases and their substrates establish the signalling networks of cells, governing all aspects of health and diseases. Due to the significant time and resource cost in experimentally demonstrating the relationship between kinases and substrates, computational methods have been key workhorses for prioritizing phosphorylation sites that are promising candidates prior to experimental verification. While many methods have been developed for predicting the cognate kinases of phosphosites, only a subset could perform kinase-specific predictions (5). Among the kinase-specific methods, most identify potential phosphorylation sites based on static information such as the amino acid sequences and features derived from them and other sources such as protein–protein interaction (PPI) databases. For example, Musite combines sequence similarity to known phosphosites with protein disorder scores (6); Predikin uses both crystal structure and molecular modelling for predicting kinase substrates (7); PhosphoPICK incorporates PPIs in their prediction procedure (8); GPS 5.0 curates experimentally identified phosphosites and uses a position weight determination regression model for prediction (9); and NetworKIN uses information from franking sequence of residues, evolutionary phylogeny and a network proximity score, based on PPIs from STRING database for kinase-substrate prediction (10).

With recent major advances in mass spectrometry-based phosphoproteomics technologies, especially with the adoption of data-independent acquisition mass spectrometry (11,12), large numbers of phosphosites can now be quantified in a single experiment (13). These phosphoproteomics data provide a rich information resource that can be used for modelling the dynamics of each phosphorylation site in cells and tissues. Yet, very few computational methods utilize quantitative phosphoproteomics data for kinase-substrate prediction (14). A few examples include CoPhosK, which uses co-phosphorylation patterns and interaction networks (15), and PUEL, an ensemble of support vector machine (SVM) models that predicts kinase substrates based on both kinase recognition motifs and phosphoproteomics dynamics (14). While comprehensive lists of kinase–substrate relationships have been curated [e.g. (16)], a key challenge in using phosphoproteomics data for kinase-substrate prediction has been the relatively small proportions of known substrates that are profiled in a phosphoproteomics dataset. Given the potential utility of phosphoproteomics in capturing the dynamics of signalling in cells, tissues and complex diseases such as metabolic diseases and cancers (17), there is a growing need and opportunity for developing advanced computational methodologies that leverage phosphoproteomics data to predict kinase–substrate relationships (18).

Here, we aim to develop advanced machine learning models for kinase-substrate prediction by addressing several key challenges in learning from large-scale phosphoproteomics datasets. Specifically, (i) to overcome the relatively small number of experimentally validated kinase substrates in a phosphoproteomics dataset, we introduce a ‘pseudo-positive’ learning strategy for increasing the size of training datasets during model building; (ii) to increase the model stability and usage of training data, we implement a data resampling-based ensemble learning strategy for classification models; and (iii) to improve the model performance, we utilize a snapshot ensemble learning strategy (19) and incorporate additional learning features extracted from amino acid sequences. To evaluate the models, we collect published large phosphoproteomics datasets. Using this collection and a panel of classification algorithms including both traditional and deep learning models, we first demonstrate the effectiveness of pseudo-positive and data resampling-based ensemble learning strategies in improving model prediction and stability. Consistent with our expectation, we show that the ensemble of deep learning models generally leads to better performance than the ensemble of traditional models (20). We next demonstrate that employing the snapshot ensemble learning techniques for creating ensemble deep learning neural networks and incorporating CKSAAP (composition of *k*-spaced amino acid pairs) learning feature leads to further improvement in model performance. We propose the resulting ensemble deep learning model, called ‘SnapKin’, as a useful method for kinase-substrate prediction.

## Materials and methods

### Phosphoproteomics data processing and learning feature extraction

Seven public phosphoproteomics datasets generated from various cell types and tissues under experimental perturbations were used for model evaluation (Table 1).

**Table 1.** The phosphoproteomics datasets used in this study for evaluating kinase-substrate prediction performance

Dataset (perturbation)	Abbreviation	# Phosphosites	# Features	Accession	Publication
C2C12 (differentiation)	C2C12	10 495	18	PXD023413	(21)
ESC (differentiation)	ESC	17 866	50	PXD010621	(2)
L1 (FGF)	L1-F	6864	14	PXD003631	(22)
L1 (insulin)	L1-I	12 110	14	NA	(23)
L1 (redox)	L1-R	17 857	26	PXD011525	(24)
Liver cell lines (insulin)	LCL	13 330	26	PXD001792	(25)
Mouse liver (insulin)	Liver	9687	93	PXD001792	(25)

Each dataset was preprocessed by phosphosite filtering, missing value imputation and batch correction using the PhosR package as described in detail in (21,26). Log_2_ fold changes relative to controls in each dataset were calculated and normalized using min–max scaling. This processed phosphoproteomics quantification was then used as learning features. To incorporate motif information, for each phosphoproteomics dataset, we scored the amino acid sequences of all phosphosites in the datasets based on the known kinase recognition motifs using the frequencyScoring function in the PhosR package (26). In particular, these motif scores convert the sequence into a numeric feature based on the frequency of amino acids appearing at each location on the sequence of a phosphosite. The number of amino acids included in the sequence window was 31 by default as output from the MaxQuant software (27). After these calculations, the motif scores were min–max scaled and combined with the phosphorylation dynamics (i.e. normalized log_2_ fold change) to form the input data for training each learning model. Furthermore, we assessed the utility of additional learning features extracted from phosphosite sequences using methods in listed in Table 2 (28). These features were subsequently combined with both the phosphorylation dynamics and the motif scores for kinase-substrate prediction. Finally, we also explored the potential utility of PPI as a learning feature. In particular, PPI scores were extracted from the STRING database (29). These scores were assigned to their respective phosphosites based on the PPI between the host proteins of phosphosites and kinases. As with prior steps, this learning feature was combined with phosphorylation dynamics and motif scores for kinase-substrate prediction. The 5-fold cross-validation was used for assessing model performance using above different combinations of learning features.

**Table 2.** Protein sequence encoding methods

Method	Abbreviation	Category	Reference
Amino acid composition	AAC	Amino acid composition	(30)
Composition of *k*-spaced amino acid pairs	CKSAAP	Amino acid composition	(31)
Grouped amino acid composition	GAAC	Grouped amino acid composition	(31)
Normalized Moreau–Broto	NMBroto	Autocorrelation	(32)
Quasi-sequence-order descriptors	QSOrder	Quasi-sequence order	(33)
Amphiphilic PAAC	APAAC	Pseudo-amino acid composition	(34)
Binary—20 bit	Binary	Residue composition	(35)

### Pseudo-positive strategy

Data augmentation is a common strategy for machine learning tasks that deal with small datasets (36). Due to the limited positive training examples in our kinase-substrate prediction task (Table 3), we propose the following steps for creating additional positive training examples [i.e. phosphosites that are curated in the PhosphoSitePlus database (37) as being phosphorylated by a specific kinase] with a matching number of negative examples (i.e. phosphosites that are not annotated as substrates of a given kinase within the PhosphoSitePlus database):

- Separate the phosphosites in the training dataset into the positive sites ($\mathcal{P}$) and the remaining sites that exclude the positive sites ($\mathcal{S} \setminus \mathcal{P}$).
- For the ${n}_{\rm p}$ phosphosites in the positive set denoted by $\mathcal{P} = \{ {{x}_1, \ldots ,{x}_{{n}_{\rm p}}} \}$, construct a list consisting of every unique pair of positive sites given by
  \begin{eqnarray*}\mathcal{F} = \left\{ {\left( {{x}_1,{x}_2} \right),\left( {{x}_1,{x}_3} \right), \ldots ,\left( {{x}_{{n}_{\rm p} - 1},{x}_{{n}_{\rm p}}} \right)} \right\}.\end{eqnarray*}
- For each pair in $\mathcal{F}$, generate a pseudo-positive site using the following equation:
  \begin{eqnarray*}{x}_{{\rm pseudo}} = \frac{{a + b}}{2},\end{eqnarray*}

where $( {a,b} ) \in \mathcal{F}$. The pseudo-positive site can then be expressed as


\begin{eqnarray*} \mathcal{P}^{\prime} = \left\{ {x_{i\ }^{\prime}{\mathrm{|}}\ x_i^{\prime} = \frac{{a + b}}{2}} \right\}, \end{eqnarray*}

where $( {a,b} ) \in \mathcal{F}$.

- The negative set $\mathcal{N}$ is a subsample of $\mathcal{S} \setminus \mathcal{P}$ of the same size as the combined number of observations in $\mathcal{P}$ and $\mathcal{P}^{\prime}$. That is, $\mathcal{N} = \{ {{x}_1, \ldots ,{x}_{{n}_{\rm n}}} \} \subseteq \mathcal{S} \setminus \mathcal{P}$, where ${n}_{\rm n} = | \mathcal{P} | + | {\mathcal{P}^{\prime}} |$.
- The final training set is then the combined positive, pseudo-positive and negative site set $\mathcal{P} \cup \mathcal{P}^{\prime} \cup \mathcal{N}$.

**Table 3.** The number of known substrates in each dataset for each kinase

Dataset	AKT1	CDK1	CDK5	CSNK2A1	GSK3B	MAPK1	MAPK14	MAPK3	MAPK8	mTOR	PRKACA	PRKCA
C2C12	17	11	7	15	15	36	19	15	8	38	19	15
ESC	21	25	10	7	18	62	20	19	10	52	22	7
L1-F	15	7	3	5	4	24	13	10	4	24	29	11
L1-R	33	15	14	11	14	80	26	37	13	49	37	24
L1-I	9	4	4	10	6	18	7	16	7	11	21	16
LCL	16	7	4	7	11	30	19	13	6	27	24	9
Liver	19	18	6	9	11	51	17	15	9	39	16	7

**Figure 1.**
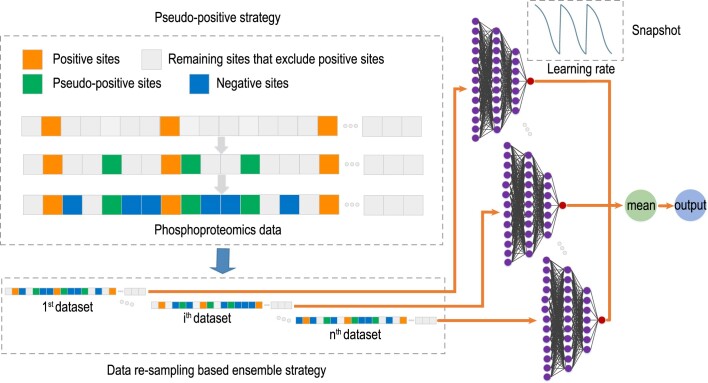
The framework of SnapKin. Schematic representation of ‘pseudo-positive’ strategy and data resampling procedure implemented in SnapKin.

This pseudo-positive strategy, schematically summarized in Figure 1, is able to generate at most ${{{n}_{\rm p}( {{n}_{\rm p} - 1} )}}/{2}$ pseudo-positives due to the possible overlap between pseudo-positive sites and positive sites, meaning the subsequent adapted training dataset uses an additional at most ${{{n}_{\rm p}( {{n}_{\rm p} - 1} )}}/{2}$ negative sites. This is particularly useful for supervised learning approaches that perform poorly with small sample sizes. Since substrates for a particular kinase typically exhibit similar temporal profiles (38), the pseudo-positive examples generated using this strategy make biological sense and have similar phosphorylation patterns to known phosphosites of a kinase, and hence can help improve the performance of the supervised learning approaches. Note that both the dynamic phosphoproteomics profile and the motif feature extracted from sequences are numeric and can be averaged for creating pseudo-positive sites.

### Data resampling-based ensemble strategy

The data resampling-based ensemble strategy is well known for its effectiveness in alleviating small sample size and has been demonstrated to enhance the robustness of the model and its generalizability to unseen data (39). To further improve the model performance in our kinase-substrate prediction task, we implement a data resampling procedure to generate multiple training datasets and compute a final prediction score from their collective predictions through model averaging. This framework involves choosing the number of models within the ensemble denoted by ${n}_{\rm e}$ (set as 10 in this study) and is implemented in the following steps:

- Separate the phosphosites in the training dataset into the positive sites ($\mathcal{P}$) and other sites that are not the positive sites ($\mathcal{S} \setminus \mathcal{P}$).
- Generate ${n}_{\rm e}$ separate training datasets denoted by ${T}_1, \ldots ,{T}_{{n}_{\rm e}},$ where each dataset ${T}_i = \mathcal{P} \cup {\mathcal{N}}_i$ involves generating a new negative set ${\mathcal{N}}_i$ by repeated subsampling from ($\mathcal{S} \setminus \mathcal{P}$), requiring $| {{\mathcal{N}}_i} | = | \mathcal{P} |$.
- For each training dataset, train a separate model ${f}_i( {x{\mathrm{|}}{T}_i} )$ for a total of ${n}_{\rm e}$ models.
- To compute the prediction of a phosphosite $x$, compute the prediction probability from each model ${f}_i$ and take the average of the prediction probabilities. Denote $F$ to be the prediction from the ensemble model. The prediction is then defined by
  \begin{eqnarray*}F\left( x \right) = \frac{1}{{{n}_{\rm e}}}\mathop \sum \limits_{i = 1}^{{n}_{\rm e}} {f}_i\left( {x{\mathrm{|}}{T}_i} \right).\end{eqnarray*}

This framework allows for an increased usage of $\mathcal{S}\backslash \mathcal{P}$ in training a model since $\big| {\bigcup\nolimits_{i = 1}^{{n}_{\rm e}} {{\mathcal{N}}_i} } \big| \ge | {{\mathcal{N}}_i} |$ for each $i$. Additionally, by also including set $\mathcal{P}^{\prime}$ in the above pseudo-positive procedure for each training set, the ensemble procedure can be in conjunction with the pseudo-positive procedure where each ${\mathcal{N}}_i$ will have a size of $| \mathcal{P} | + | {\mathcal{P}^{\prime}} |$.

### Classification models

We implemented a variety of classification models for testing their performance on kinase-substrate prediction. These include five traditional models and a deep learning model. For the traditional models, we implemented naive Bayes (NB), fitted using the discrim R package; logistic regression (LR) using the glm R package; SVM with a radial basis function using the kernlab R package; random forest (RF) with 500 trees using the ranger R package; and XGBoost (XG) with 1000 trees using the xgboost R package. For the deep learning model, we implemented a densely connected neural network (DNN) where we used fully connected neurons with hidden neurons activated by ‘Leaky Relu’ function and output neurons activated by a ‘Sigmoid’ function. We found the hidden layers of three to be sufficient and determined the width of each layer using the following heuristic rules. We predefined the widths of the DNN as 2, 4, 8, 16, 32, 64 and 128, and the first hidden layer of the DNN has a width equal to the largest value in the predefined width and less than or equal to the initial input features. Then, it decreases by halving the width until the number of layers (i.e. 3) is reached. Other hyperparameters in our DNN include the ADAM optimizer (40), the binary cross-entropy loss function, epochs (150), learning rates of 0.001, 0.01 or 0.1, and batch sizes of 32 or 64 obtained from a nested cross-validation of each fold.

### Implementation of SnapKin

The SnapKin model adopts the same architecture as in the above DNN but uses the stochastic gradient descent and a learning rate scheduler (19) defined as follows:


\begin{eqnarray*}{\mathrm{\lambda }}\left( t \right) = \frac{{{{\mathrm{\lambda }}}_0}}{2}\left( {\cos \left( {\frac{{{\mathrm{\pi }}\,{\rm mod}\left( {t - 1,T/M} \right)}}{{T/M}}} \right) + 1} \right),\end{eqnarray*}

where ${{\mathrm{\lambda }}}_0$ is the initial learning rate (set as 0.01), $t$ is the iteration number, $T$ is the total number of training iterations (set as 1000) and $M$ is the number of snapshots of the DNN (set as 10 in this study to match the ensemble of DNNs).

In addition, SnapKin adopts both pseudo-positive and data resampling learning strategies. Note that similar to the model ensemble strategy described above, a subsampling of the unannotated sites in a given dataset is performed to generate a training set ${T}_i = \mathcal{P} \cup \mathcal{P}^{\prime} \cup {\mathcal{N}}_i$ prior to training ($i = 1$) and after each snapshot is taken ($i = 2, \ldots ,M$) and therefore enables better usage of data without introducing further computational time and model complexity, allowing our modification adhere to the ‘train 1, get *M* for free’ spirit of the original snapshot ensemble algorithm.

### Model evaluation

We applied a stratified *k*-fold cross-validation procedure for evaluating model performance. Specifically, we used *k* = 5 in this study and repeated the cross-validation process 50 times to quantify the variability of model predictions. By stratifying each fold of the data, we ensure, for a given kinase, each fold maintains the ratio of positive and negative phosphosites in the original dataset. Each method was evaluated on each test fold of each phosphoproteomics dataset using the precision–recall (PR) curve defined by the four quantities: true positive (TP), phosphosites that are annotated to a specific kinase in the dataset; true negative (TN), phosphosites in the dataset not annotated to a specified kinase; false positive (FP), unannotated phosphosites that were predicted as a kinase substrate; and false negative (FN), known kinase substrates that are predicted as not a substrate of that kinase. A PR curve is commonly used for comparing model performance especially when the dataset is highly imbalanced (41). It is a trade-off between


\begin{eqnarray*}{\rm precision}\left( t \right) = \frac{{{\rm TP}\left( p \right)}}{{{\rm TP}\left( p \right) + {\rm FP}\left( p \right)}}\end{eqnarray*}

and


\begin{eqnarray*}{\rm recall}\left( p \right) = \frac{{{\rm TP}\left( p \right)}}{{{\rm TP}\left( p \right) + {\rm FN}\left( p \right)}},\end{eqnarray*}

where $p$ is the prediction threshold from each classifier. While the PR curves provide a threshold-based comparison of models, we also used the areas under the PR curves as summaries and averaged them across all test folds in the cross-validation for quantifying the overall performance of each model on each phosphoproteomics dataset. This allows us to easily compare models using statistical testing. Specifically, we used a one-sided Wilcox rank sum test with the hypotheses that (i) ${H}_{{\rm a}1}$: pseudo-positive strategy improves prediction of single models; (ii) ${H}_{{\rm a}2}$: ensemble learning improves prediction of single models; and (iii) ${H}_{{\rm a}3}$: ensemble learning in conjunction with pseudo-positive improves prediction on a single model trained with pseudo-positive strategy. The areas under the PR curves from the 50 repeated runs of the 5-fold cross-validation were used as the primary statistics to compute the significance.

Finally, we used the standard deviation in the areas under the PR curves from the 50 runs of the 5-fold cross-validation to quantify the stability of the models. We then tested whether the standard deviation from using ensemble learning is significantly smaller than single models across the seven phosphoproteomics datasets.

### Characterizing SnapKin predictions on muscle differentiation and inhibition phosphoproteomics datasets

To assess the performance of SnapKin, we first evaluated the substrate prediction of mTOR and MAPK1 using the time-course muscle differentiation phosphoproteome dataset, and then evaluated the predicted MAPK1 substrates using the MAPK1 inhibition muscle differentiation phosphoproteome dataset. The time-course differentiation data were generated during a 5-day differentiation, which includes four time points (0, 30 min, 24 h and 5 days). The inhibition phosphoproteome data were collected on day 3 of differentiation with (control) and without MAPK1 inhibitor. We used iceLogo (42), a visualization tool for conserved patterns in protein and nucleotide sequences, to generate consensus motifs from SnapKin-predicted substrates (>0.8) for MAPK1 and mTOR, respectively. The SnapKin-predicted substrates for MAPK1 and mTOR were visualized for their temporal profiles using $z$-score standardized log_2_ fold change of phosphorylation compared to the zero time point. The known substrates derived from PhosphoSitePlus and SnapKin-predicted substrates for MAPK1 were also visualized for their inhibition profiles using $z$-score standardized log_2_ fold change of phosphorylation compared to the control group.

### Benchmarking with existing methods

We benchmarked SnapKin with other four previously published kinase-substrate prediction methods, including NetworKIN (10), GPS 5.0 (9), PhosphoPICK (8) and CoPhosK+ (15). Among these, NetworKIN, GPS 5.0 and PhosphoPICK rely primarily on static information like sequences and PPIs; the same dataset cannot be directly applied to train each model. To this end, we derived kinase-substrate prediction scores of 12 kinases for each of the above three methods from a kinase-substrate prediction resource (43), and we run the CoPhosK+ pipeline by inputting each of 7 phosphoproteomics datasets and obtained the prediction score of each phosphosite in the datasets of 12 kinases. We then obtained the prediction scores from all methods on the overlapped phosphosites. Phosphosites were scale-ranked based on their scores derived from each method:


\begin{eqnarray*}{\rm scaled}\ {\rm rank}\left( x \right) = \frac{{{\rm rank}\left( {{\rm prediction}\ {\rm score}\left( x \right)} \right)}}{n},\end{eqnarray*}

where $n$ denotes the number of phosphosites that are common in all methods. Since mTOR was not included in NetworKIN predictions, NetworKIN was excluded from the mTOR prediction comparison.

## Results

Here, we present the findings on using pseudo-positive and data resampling-based ensemble learning strategies (Figure 1) for improving model prediction and stability. Classification models included in the evaluation are NB, LR, SVM, RF, XG and DNN. Given that most supervised learning approaches rely on and generally perform better with more training examples, we first assessed the utility of proposed learning strategies and features using MAPK1 and mTOR, the two kinases with overall the most quantified substrates across the datasets based on the known kinase-substrate annotation in PhosphoSitePlus database (Table 3). We subsequently benchmarked the performance of the proposed SnapKin model with other alternative methods for predicting substrates of kinases that have more than two known substrates across all datasets. Lastly, we analyse the predictions from SnapKin on the muscle cell phosphoproteomics datasets, providing literature support for putative candidates uncovered by this computational model.

### Pseudo-positive strategy improves model prediction

A key limitation of using supervised learning models for kinase-substrate prediction is the lack of high-quality positive training examples, owing to the small number of experimentally validated substrates for the majority of known kinases (44). Despite numerous phosphosites identified in phosphoproteomics studies, only a fraction serve as negative training examples due to classification model sensitivities to class imbalance (45). Since the substrates often show similar patterns of changes in phosphorylation upon the perturbation of their responsive kinases (e.g. stimulation, inhibition, differentiation) (38), we introduce a simple strategy to generate ‘pseudo-positive’ examples by averaging phosphorylation profiles of known substrate pairs for each kinase. This approach adheres closely to data augmentation commonly used in machine learning tasks for learning from small datasets (36).

**Figure 2.**
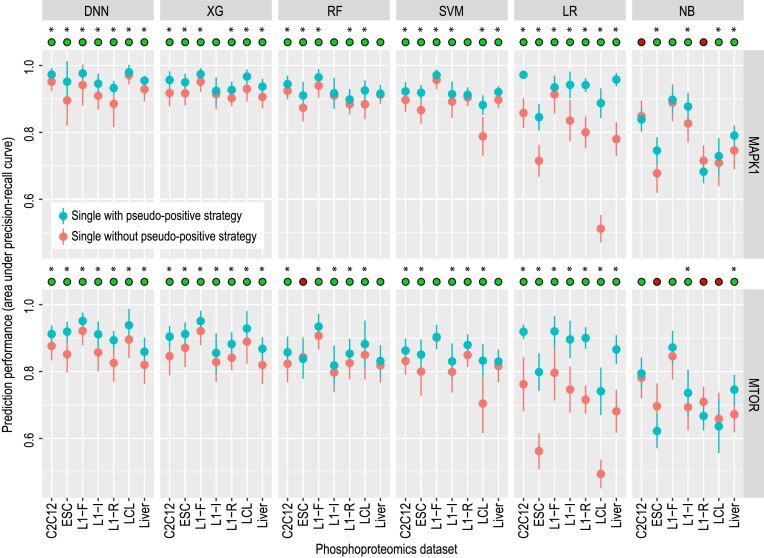
Prediction performance assessment of models with and without using the pseudo-positive learning strategy across the seven phosphoproteomics datasets. Solid dots represent the mean performance of each model from a 5-fold cross-validation and error bars represent the standard deviation from 50 repeated trials of the 5-fold cross-validation. The green circles on top of each panel denote the cases when using the pseudo-positive strategy improves model performance and the red circles denote the opposite. * denotes *P* < 0.05 using a one-sided Wilcox rank sum test.

The utility of these pseudo-positive examples can be assessed by evaluating the prediction performance of models on test datasets using cross-validation. Figure 2 summarizes the prediction performance of each model with and without the use of pseudo-positive examples. Raw numeric results are included in Supplementary Table S1. Except for a few results in RF and NB classifiers, we found that the use of pseudo-positive examples resulted in significantly improved model performance in terms of area under the PR curve on both the substrates of MARK1 and mTOR. Especially, we observed as high as 40% accuracy gain with the ‘pseudo-positive strategy’ with the LR classifier on dataset LCL. Note that similar conclusions can be obtained with other classifiers and datasets. These results demonstrate that the pseudo-positive strategy is effective for improving prediction across a range of classification models.

### Data resampling-based ensemble improves model prediction and stability

**Figure 3.**
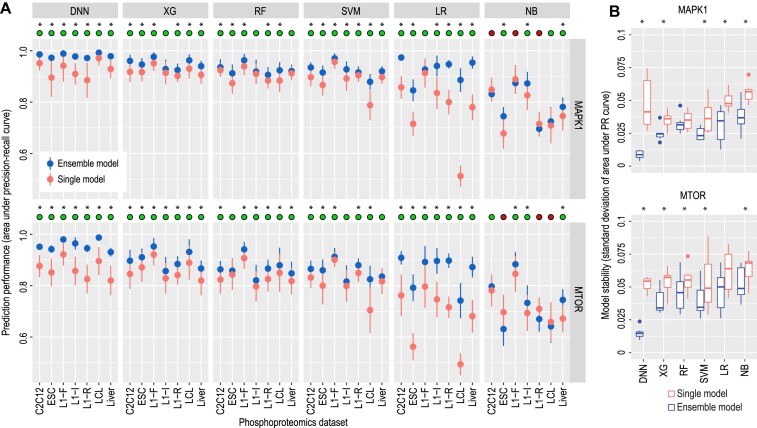
Performance and stability analysis of models with and without a data resampling ensemble learning strategy across the seven phosphoproteomics datasets. (**A**) Solid dots represent the mean performance of each model from a 5-fold cross-validation and error bars represent the standard deviation from 50 repeated trials of the 5-fold cross-validation. The green circles on top of each panel denote the cases when using the ensemble strategy improves model performance and the red circles denote the opposite. (**B**) Stability comparison between single and ensemble models using the data resampling ensemble strategy. Stability is measured by the standard deviation of areas under the PR curves from the 50 repeated 5-fold cross-validation trials, with blue and orange boxplots representing the model results with and without this strategy, respectively. * denotes *P* < 0.05 using a one-sided Wilcox rank sum test.

The data resampling-based ensemble strategy has been shown to be effective when learning from data with sample training examples and can improve model stability and generalizability (39). To this end, we propose a data resampling-based ensemble learning strategy that involves generating multiple training datasets and consequently fitting multiple independent models in order to determine a collective prediction (39). In our kinase-substrate prediction setting, the motivation for the data resampling-based ensemble learning stems from the need to utilize more of the negative training examples, given the large number of phosphosites quantified in the phosphoproteomics experiments, and the assumption that the majority of these are not substrates of a given kinase. We compared the performance of models trained with and without using the data resampling-based ensemble learning strategy and found in most cases a significant improvement in prediction is achieved when the model is trained using the ensemble learning strategy (Figure 3A, Supplementary Table S2). Another key advantage of ensemble learning is its robustness to data noise, which can lead to more stable and reproducible predictions (20). Indeed, by comparing the variability in model prediction from the 50 repeated runs of the 5-fold cross-validation, we observed a reduction of variance in most cases across the six models when the data resampling-based ensemble strategy is used (Figure 3B).

**Figure 4.**
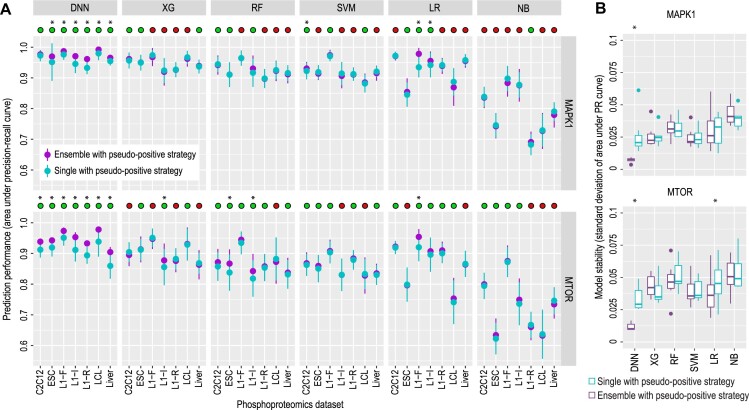
Performance and stability analysis of single and ensemble models utilizing the pseudo-positive strategy across seven phosphoproteomics datasets. (**A**) Solid dots represent the mean performance of each model and error bars represent the standard deviation from 50 repeated runs of the 5-fold cross-validation. The green circles on top of each panel denote the cases when using the ensemble strategy improves model performance and the red circles denote the opposite. (**B**) Stability analysis for single and ensemble models using the pseudo-positive strategy. Purple and light blue boxplots represent models with and without the data resampling ensemble strategy, respectively. Stability is measured by the standard deviation of areas under the PR curves from the 50 repeated 5-fold cross-validation trials, with each box reflecting data from all seven phosphoproteomics datasets. * denotes *P* < 0.05 using a one-sided Wilcox rank sum test.

Furthermore, the data resampling-based ensemble strategy can be used in conjunction with the pseudo-positive learning strategy and may further improve model performance. To this end, we compared the prediction performance and stability of models trained using pseudo-positive examples and with or without using the ensemble learning strategy. While the traditional classification models show no ‘synergistic’ improvement from using both learning strategies, we found additional improvement for the deep learning model of DNN on both model prediction (Figure 4A, Supplementary Table S3) and stability (Figure 4B). These results are in line with the higher model complexity/flexibility of DNNs compared to traditional models, which may allow them to benefit more from additional training data.

### Developing and benchmarking SnapKin for kinase-substrate prediction

**Figure 5.**
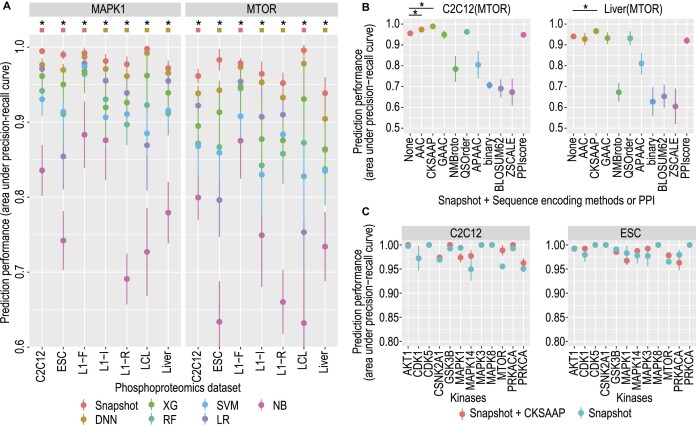
Performance evaluation of the snapshot ensemble and integration of sequence features. (**A**) Snapshot ensemble performance comparision. All models employ pseudo-positive and ensemble learning strategies, except the snapshot model, which integrates both pseudo-positive and data resampling approaches in each DNN snapshot. Solid dots represent the mean performance and error bars represent the standard deviation from 50 repeated trials of the 5-fold cross-validation. Red squares denote when the standard deviation of the snapshot model is smaller than the second-best method (in all cases, DNN), whereas brown squares denote otherwise. * denotes *P* < 0.05 comparing snapshot with the second-best method using a one-sided Wilcox rank sum test. (**B**, **C**) Model performance using sequence encoding or PPIs. (**B**) Performance comparison of the snapshot model, with or without integrating features from various sequence encoding methods or PPI scores, for mTOR in both C2C12 and liver datasets. * denotes *P* < 0.05 comparing the snapshot model with other methods using a one-sided Wilcox rank sum test. (**C**) Performance comparison of the snapshot model with or without using features derived from CKSAAP across 12 kinases for both C2C12 and ESC datasets.

Our results from the above evaluation indicate that pseudo-positive and data resampling-based ensemble learning strategies are effective in improving model prediction and stability. They also demonstrate the competitive performance of the deep learning model (DNN) compared to traditional models, especially when used together with the two proposed learning strategies where additional performance gain is achieved mostly on DNN only. To further optimize model performance, we next introduced snapshot ensemble (19) wherein the pseudo-positive and data resampling strategies are incorporated into a snapshot ensemble model. When compared to other models trained using pseudo-positive in conjunction with the ensemble learning, using snapshot shows the best overall prediction performance across all seven phosphoproteomics datasets and comparably small variability to the second-best model (Figure 5A). Since in all cases the second-best method is DNN (trained using pseudo-positive and ensemble learning), which already has the smallest variability compared to traditional classification models (Figure 4B), these results suggest that the snapshot approach achieves the best prediction performance without losing model stability compared to DNN. Given that in our implementation the DNN and snapshot approach use the same network architecture, the performance improvement of the snapshot approach compared to DNN indicates that the snapshot ensemble brings further benefit on creating ensemble deep learning models in which various near-optimal models are extracted and combined in a single training process (46).

Previous studies summarized different categories of methods for extracting information from amino acid sequences (28). To this end, we next delved into methods covering each category and found that the CKSAAP encoding technique yielded the best performance in both the C2C12 and liver datasets (Figure 5B) and this encoding method consistently improved or matched the performance across most tested kinases (Figure 5C). We also evaluated the model performance with PPI as additional learning features and found that this did not lead to an improvement in model prediction accuracy (Figure 5B). This could be due to the transient nature of kinase–substrate interactions that may not be captured by PPIs. We therefore included CKSAAP as an additional feature in our model to form ‘SnapKin’ for subsequent analysis.

**Figure 6.**
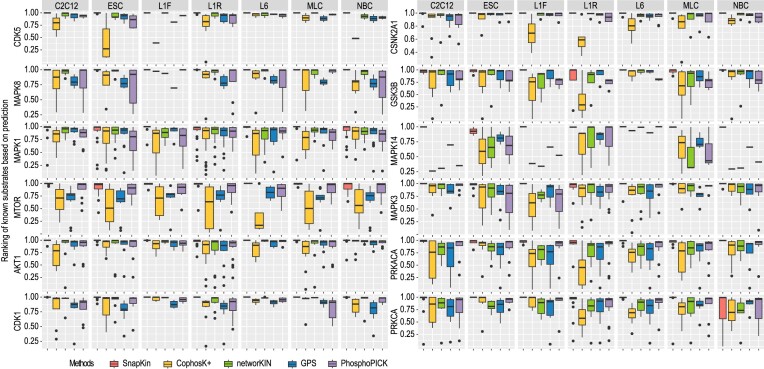
Evaluation of SnapKin prediction performance. Comparison of prediction performance of SnapKin and other kinase-substrate predictive algorithms, including CoPhosK+, NetworKIN, GPS and PhosphoPICK, across the 7 phosphoproteomics datasets and 12 kinases.

Lastly, we then benchmarked SnapKin with existing prediction methods, including NetworKIN, GPS 5.0, PhosphoPICK and CoPhosK+, for each of the 7 phosphoproteomics datasets and for each of 12 kinases. While NetworKIN, GPS 5.0 and PhosphoPICK rely primarily on sequence and structural information around the residue, including evolutionary, network and PPI information extracted from databases and curated from the literature, CoPhosK+ uses both static motif features and information extracted from dynamic phosphoproteomics data. This provides an informative comparison with our method that uses dynamic phosphorylation profiles besides the sequence information. We found that the ranking of known substrates of all 12 tested kinases based on the prediction score from SnapKin was generally higher compared to the other four methods in the majority of datasets (Figure 6). These results suggest that SnapKin in general outperforms other methods for kinase-substrate prediction.

### SnapKin kinase-substrate predictions on the muscle phosphoproteomics dataset

**Figure 7.**
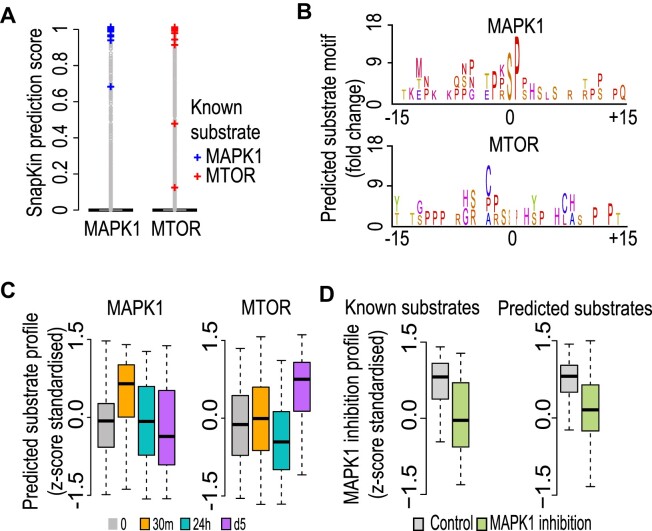
Muscle phosphoproteomics data analysis. (**A**) SnapKin prediction score on profiled phosphosites in the C2C12 differentiation dataset. Known MAPK1 (*n* = 36) and mTOR substrates (*n* = 38) are highlighted in blue and red, respectively. (**B**) The consensus motif generated from the top SnapKin-predicted MAPK1 and mTOR substrates. (**C**) Phosphorylation profiles of the C2C12 differentiation phosphoproteome derived from SnapKin-predicted MAPK1 and mTOR substrates, where ‘putative substrates’ are those 100 top-ranked based on prediction scores. (**D**) Phosphorylation profiles of known MAPK1 (*n* = 44) and the top 100 SnapKin-predicted MAPK1 substrates in the C2C12 inhibition phosphoproteome.

We next characterized the prediction results from SnapKin on the C2C12 differentiation phosphoproteomics dataset. We found that while most of the known MAPK1 and mTOR substrates have high prediction scores, the majority of the phosphosites in the dataset have close to zero prediction scores (Figure 7A), consistent with the high selectivity of many kinases on their substrates (47). For the top 100 putative MAPK1 and mTOR substrates predicted by SnapKin, the two groups show similar proline-directed consensus motifs (Figure 7B), which are consistent with known MAPK1 and mTOR recognition motifs and common among many other kinases. Nevertheless, the phosphorylation profiles clearly distinguish the two groups with putative MAPK1 substrates showing acute phosphorylation increase at 30 min time point and those of mTOR showing much slower response at day 5 (Figure 7C). These results demonstrate that kinase recognition motifs alone may not be sufficient to identify kinase substrates and the phosphorylation profiles can be highly informative in distinguishing kinase substrates that share similar motifs. Furthermore, we characterized the MAPK1 substrates upon MAPK1 inhibition during C2C12 differentiation (21). We found that compared to control samples, both MAPK1 known substrates and putative substrates showed a reduction of phosphorylation level (Figure 7D). Indeed, several identified putative substrates, such as SPEG (48), PAK1 (49) and SORBS2 (50), have already been linked to muscle development. These findings highlight the potential use of SnapKin for prioritizing kinase-substrate prediction for downstream experimental validations.

## Discussion

Global phosphoproteomics studies provide an unprecedented opportunity to characterize signalling networks in health and diseases (51). While machine learning methods and especially deep learning algorithms can benefit from the abundant data generated from such studies, phosphoproteomics data-specific characteristics create various computational challenges limiting their direct application. One particular issue is the class imbalance caused by the small number of known kinase–substrate relationships because, compared to a small set of positive examples of a kinase, significantly more phosphosites can be used as negative examples for model training (45). Since most prediction models are sensitive to class imbalance, in this study, we have proposed various computational strategies to increase the size of the training dataset without introducing class imbalance. Nevertheless, other computational strategies such as cost-sensitive learning (52), which has been used for training classical neural networks (53), could be explored for developing ensemble deep learning models that alleviate the limit set by class imbalance, and may allow significantly more phosphosites to be included in training prediction models.

Typically, prediction models need to be trained using both positive and negative examples. For a kinase, although the positive examples can be found from known substrates such as those annotated in PhosphoSitePlus database (37), the negative examples have to be defined independently as such information is often not available. Because only a relatively small number of phosphosites may be phosphorylated by each kinase owing to kinase-substrate selectivity (47), we treated the subsampled phosphosites that exclude the positive examples as negative examples, given that the chance of including unknown positive sites is small. While this assumption may have minimum effect on the comparison of model performance, including additional learning procedures that can take into account uncertainty in sampling negative examples may provide a more precise estimate of model accuracy (54) and will be explored in future work. Related to this, although the positive examples can be curated using known kinase substrates from an annotation database such as PhosphoSitePlus, there are various other databases [e.g. Phospho.ELM (55) and PhosphoPOINT (56)] that can be used for such a purpose as well and the quality of the annotations may be dependent on the types of validation experiments and the biological systems in which they are validated. Developing methods that can take into consideration the type of evidence in kinase-substrate validation and the potential false positive examples in these data sources during model training will likely lead to further improvements in prediction accuracy.

In its current implementation, SnapKin only takes single phosphoproteomics data for kinase-substrate prediction. A future direction of SnapKin extension is to learn from multiple phosphoproteomics data so as to improve the confidence of prediction results while also reducing the potential of model overfitting. In particular, the ensemble learning framework used can facilitate such an extension by using different models each learning from a different phosphoproteomics dataset. Finally, although experimental evaluation of kinase substrates remains time consuming and labour intensive, significant efforts have been made with the systematic mapping of kinase and their downstream substrates (57). Such experimental data resources will not only help validate putative kinase substrate candidates from computational predictions but also lead to the improved predictive accuracy of computational models as the increasing number of experimentally validated kinase substrates will enable an increasingly larger data repertoire to be curated for training computational models.

## Supplementary Material

lqad099_Supplemental_Files

## Data Availability

SnapKin’s source code is available in Zenodo at https://doi.org/10.5281/zenodo.10038862. All phosphoproteomics datasets analysed in this study are published previously and their publications and accessions are listed in Table 1.

## References

[1] Humphrey S.J. , James D.E., Mann M. Protein phosphorylation: a major switch mechanism for metabolic regulation. Trends Endocrinol. Metab. 2015; 26:676–687. 26498855 10.1016/j.tem.2015.09.013

[2] Yang P. , Humphrey S.J., Cinghu S., Pathania R., Oldfield A.J., Kumar D., Perera D., Yang J.Y.H., James D.E., Mann M. et al. Multi-omic profiling reveals dynamics of the phased progression of pluripotency. Cell Syst. 2019; 8:427–445. 31078527 10.1016/j.cels.2019.03.012 PMC6544180

[3] Swaffer M.P. , Jones A.W., Flynn H.R., Snijders A.P., Nurse P. CDK substrate phosphorylation and ordering the cell cycle. Cell. 2016; 167:1750–1761. 27984725 10.1016/j.cell.2016.11.034 PMC5161751

[4] Emdal K.B. , Palacio-Escat N., Wigerup C., Eguchi A., Nilsson H., Bekker-Jensen D.B., Rönnstrand L., Kazi J.U., Puissant A., Itzykson R. et al. Phosphoproteomics of primary AML patient samples reveals rationale for AKT combination therapy and p53 context to overcome selinexor resistance. Cell Rep. 2022; 40:111177. 35947955 10.1016/j.celrep.2022.111177 PMC9380259

[5] Blom N. , Gammeltoft S., Brunak S. Sequence and structure-based prediction of eukaryotic protein phosphorylation sites. J. Mol. Biol. 1999; 294:1351–1362. 10600390 10.1006/jmbi.1999.3310

[6] Gao J. , Thelen J.J., Dunker A.K., Xu D. Musite, a tool for global prediction of general and kinase-specific phosphorylation sites. Mol. Cell. Proteomics. 2010; 9:2586–2600. 20702892 10.1074/mcp.M110.001388 PMC3101956

[7] Saunders N.F.W. , Kobe B. The Predikin webserver: improved prediction of protein kinase peptide specificity using structural information. Nucleic Acids Res. 2008; 36:W286–W290. 18477637 10.1093/nar/gkn279 PMC2447752

[8] Patrick R. , Lê Cao K.-A., Kobe B., Bodén M. PhosphoPICK: modelling cellular context to map kinase-substrate phosphorylation events. Bioinformatics. 2015; 31:382–389. 25304781 10.1093/bioinformatics/btu663

[9] Wang C. , Xu H., Lin S., Deng W., Zhou J., Zhang Y., Shi Y., Peng D., Xue Y. GPS 5.0: an update on the prediction of kinase-specific phosphorylation sites in proteins. Genomics Proteomics Bioinformatics. 2020; 18:72–80. 32200042 10.1016/j.gpb.2020.01.001 PMC7393560

[10] Horn H. , Schoof E.M., Kim J., Robin X., Miller M.L., Diella F., Palma A., Cesareni G., Jensen L.J., Linding R. KinomeXplorer: an integrated platform for kinome biology studies. Nat. Methods. 2014; 11:603–604. 24874572 10.1038/nmeth.2968

[11] Gao E. , Li W., Wu C., Shao W., Di Y., Liu Y. Data-independent acquisition-based proteome and phosphoproteome profiling across six melanoma cell lines reveals determinants of proteotypes. Mol. Omics. 2021; 17:413–425. 33728422 10.1039/d0mo00188k PMC8205956

[12] Salovska B. , Gao E., Müller-Dott S., Li W., Cordon C.C., Wang S., Dugourd A., Rosenberger G., Saez-Rodriguez J., Liu Y. Phosphoproteomic analysis of metformin signaling in colorectal cancer cells elucidates mechanism of action and potential therapeutic opportunities. Clin. Transl. Med. 2023; 13:e1179. 36781298 10.1002/ctm2.1179 PMC9925373

[13] Humphrey S.J. , Karayel O., James D.E., Mann M. High-throughput and high-sensitivity phosphoproteomics with the EasyPhos platform. Nat. Protoc. 2018; 13:1897–1916. 30190555 10.1038/s41596-018-0014-9

[14] Yang P. , Humphrey S.J., James D.E., Yang Y.H., Jothi R. Positive-unlabeled ensemble learning for kinase substrate prediction from dynamic phosphoproteomics data. Bioinformatics. 2016; 32:252–259. 26395771 10.1093/bioinformatics/btv550 PMC4739180

[15] Ayati M. , Wiredja D., Schlatzer D., Maxwell S., Li M., Koyutürk M., Chance M.R. CoPhosK: a method for comprehensive kinase substrate annotation using co-phosphorylation analysis. PLoS Comput. Biol. 2019; 15:e1006678. 30811403 10.1371/journal.pcbi.1006678 PMC6411229

[16] Chen M. , Zhang W., Gou Y., Xu D., Wei Y., Liu D., Han C., Huang X., Li C., Ning W. et al. GPS 6.0: an updated server for prediction of kinase-specific phosphorylation sites in proteins. Nucleic Acids Res. 2023; 51:W243–W250. 37158278 10.1093/nar/gkad383 PMC10320111

[17] Xiao D. , Kim H.J., Pang I., Yang P. Functional analysis of the stable phosphoproteome reveals cancer vulnerabilities. Bioinformatics. 2022; 38:1956–1963. 35015814 10.1093/bioinformatics/btac015 PMC9113330

[18] Xiao D. , Chen C., Yang P. Computational systems approach towards phosphoproteomics and their downstream regulation. Proteomics. 2023; 23:2200068. 10.1002/pmic.202200068 35580145

[19] Huang G. , Li Y., Pleiss G., Liu Z., Hopcroft J.E., Weinberger K.Q. Snapshot ensembles: train 1, get *M* for free. 2017; arXiv doi:01 April 2017, preprint: not peer reviewed https://arxiv.org/abs/1704.00109.

[20] Cao Y. , Geddes T.A., Yang J.Y.H., Yang P. Ensemble deep learning in bioinformatics. Nat. Mach. Intell. 2020; 2:500–508.

[21] Xiao D. , Caldow M., Kim H.J., Blazev R., Koopman R., Manandi D., Parker B.L., Yang P. Time-resolved phosphoproteome and proteome analysis reveals kinase signaling on master transcription factors during myogenesis. iScience. 2022; 25:104489. 35721465 10.1016/j.isci.2022.104489 PMC9198430

[22] Minard A.Y. , Tan S.-X., Yang P., Fazakerley D.J., Domanova W., Parker B.L., Humphrey S.J., Jothi R., Stöckli J., James D.E. mTORC1 is a major regulatory node in the FGF21 signaling network in adipocytes. Cell Rep. 2016; 17:29–36. 27681418 10.1016/j.celrep.2016.08.086 PMC6485955

[23] Humphrey S.J. , Yang G., Yang P., Fazakerley D.J., Stöckli J., Yang J.Y., James D.E. Dynamic adipocyte phosphoproteome reveals that Akt directly regulates mTORC2. Cell Metab. 2013; 17:1009–1020. 23684622 10.1016/j.cmet.2013.04.010 PMC3690479

[24] Su Z. , Burchfield J.G., Yang P., Humphrey S.J., Yang G., Francis D., Yasmin S., Shin S.-Y., Norris D.M., Kearney A.L. et al. Global redox proteome and phosphoproteome analysis reveals redox switch in Akt. Nat. Commun. 2019; 10:5486. 31792197 10.1038/s41467-019-13114-4 PMC6889415

[25] Humphrey S.J. , Azimifar S.B., Mann M. High-throughput phosphoproteomics reveals *in vivo* insulin signaling dynamics. Nat. Biotechnol. 2015; 33:990–995. 26280412 10.1038/nbt.3327

[26] Kim H.J. , Kim T., Hoffman N.J., Xiao D., James D.E., Humphrey S.J., Yang P. PhosR enables processing and functional analysis of phosphoproteomic data. Cell Rep. 2021; 34:108771. 33626354 10.1016/j.celrep.2021.108771

[27] Cox J. , Mann M. MaxQuant enables high peptide identification rates, individualized p.p.b.-range mass accuracies and proteome-wide protein quantification. Nat. Biotechnol. 2008; 26:1367–1372. 19029910 10.1038/nbt.1511

[28] Chen Z. , Zhao P., Li C., Li F., Xiang D., Chen Y.-Z., Akutsu T., Daly R.J., Webb G.I., Zhao Q. et al. *iLearnPlus*: a comprehensive and automated machine-learning platform for nucleic acid and protein sequence analysis, prediction and visualization. Nucleic Acids Res. 2021; 49:e60. 33660783 10.1093/nar/gkab122 PMC8191785

[29] Szklarczyk D. , Gable A.L., Nastou K.C., Lyon D., Kirsch R., Pyysalo S., Doncheva N.T., Legeay M., Fang T., Bork P. et al. The STRING database in 2021: customizable protein–protein networks, and functional characterization of user-uploaded gene/measurement sets. Nucleic Acids Res. 2021; 49:D605–D612. 33237311 10.1093/nar/gkaa1074 PMC7779004

[30] Bhasin M. , Raghava G.P.S. Classification of nuclear receptors based on amino acid composition and dipeptide composition. J. Biol. Chem. 2004; 279:23262–23266. 15039428 10.1074/jbc.M401932200

[31] Chen K. , Jiang Y., Du L., Kurgan L. Prediction of integral membrane protein type by collocated hydrophobic amino acid pairs. J. Comput. Chem. 2009; 30:163–172. 18567007 10.1002/jcc.21053

[32] Horne D.S. Prediction of protein helix content from an autocorrelation analysis of sequence hydrophobicities. Biopolymers. 1988; 27:451–477. 3359010 10.1002/bip.360270308

[33] Chou K.-C. Prediction of protein subcellular locations by incorporating quasi-sequence-order effect. Biochem. Biophys. Res. Commun. 2000; 278:477–483. 11097861 10.1006/bbrc.2000.3815

[34] Chou K.-C. Prediction of protein cellular attributes using pseudo-amino acid composition. Proteins Struct. Funct. Bioinform. 2001; 43:246–255. 10.1002/prot.1035 11288174

[35] Chen Z. , Chen Y.-Z., Wang X.-F., Wang C., Yan R.-X., Zhang Z. Prediction of ubiquitination sites by using the composition of *k*-spaced amino acid pairs. PLoS One. 2011; 6:e22930. 21829559 10.1371/journal.pone.0022930 PMC3146527

[36] Moreno-Barea F.J. , Jerez J.M., Franco L. Improving classification accuracy using data augmentation on small data sets. Expert Syst. Appl. 2020; 161:113696.

[37] Hornbeck P.V. , Kornhauser J.M., Tkachev S., Zhang B., Skrzypek E., Murray B., Latham V., Sullivan M. PhosphoSitePlus: a comprehensive resource for investigating the structure and function of experimentally determined post-translational modifications in man and mouse. Nucleic Acids Res. 2012; 40:D261–D270. 22135298 10.1093/nar/gkr1122 PMC3245126

[38] Yang P. , Zheng X., Jayaswal V., Hu G., Yang J.Y.H., Jothi R. Knowledge-based analysis for detecting key signaling events from time-series phosphoproteomics data. PLoS Comput. Biol. 2015; 11:e1004403. 26252020 10.1371/journal.pcbi.1004403 PMC4529189

[39] Yang P. , Yang Y.H., Zhou B.B., Zomaya A.Y. A review of ensemble methods in bioinformatics. Curr. Bioinform. 2010; 5:296–308.

[40] Kingma D.P. , Ba J. Adam: a method for stochastic optimization. 2015; arXiv doi:30 January 2017, preprint: not peer reviewed https://arxiv.org/abs/1412.6980.

[41] Saito T. , Rehmsmeier M. The precision–recall plot is more informative than the ROC plot when evaluating binary classifiers on imbalanced datasets. PLoS One. 2015; 10:e0118432. 25738806 10.1371/journal.pone.0118432 PMC4349800

[42] Colaert N. , Helsens K., Martens L., Vandekerckhove J., Gevaert K. Improved visualization of protein consensus sequences by iceLogo. Nat. Methods. 2009; 6:786–787. 19876014 10.1038/nmeth1109-786

[43] Xue B. , Jordan B., Rizvi S., Naegle K.M. KinPred: a unified and sustainable approach for harnessing proteome-level human kinase-substrate predictions. PLoS Comput. Biol. 2021; 17:e1008681. 33556051 10.1371/journal.pcbi.1008681 PMC7895412

[44] Needham E.J. , Parker B.L., Burykin T., James D.E., Humphrey S.J. Illuminating the dark phosphoproteome. Sci. Signal. 2019; 12:eaau8645. 30670635 10.1126/scisignal.aau8645

[45] Yang P. , Yoo P.D., Fernando J., Zhou B.B., Zhang Z., Zomaya A.Y. Sample subset optimization techniques for imbalanced and ensemble learning problems in bioinformatics applications. IEEE Trans. Cybern. 2014; 44:445–455. 24108722 10.1109/TCYB.2013.2257480

[46] Yu L. , Liu C., Yang J.Y.H., Yang P. Ensemble deep learning of embeddings for clustering multimodal single-cell omics data. Bioinformatics. 2023; 39:btad382. 37314966 10.1093/bioinformatics/btad382 PMC10287920

[47] Miller C.J. , Turk B.E. Homing in: mechanisms of substrate targeting by protein kinases. Trends Biochem. Sci. 2018; 43:380–394. 29544874 10.1016/j.tibs.2018.02.009 PMC5923429

[48] Agrawal P.B. , Pierson C.R., Joshi M., Liu X., Ravenscroft G., Moghadaszadeh B., Talabere T., Viola M., Swanson L.C., Haliloğlu G. et al. SPEG interacts with myotubularin, and its deficiency causes centronuclear myopathy with dilated cardiomyopathy. Am. J. Hum. Genet. 2014; 95:218–226. 25087613 10.1016/j.ajhg.2014.07.004 PMC4129406

[49] Joseph G.A. , Lu M., Radu M., Lee J.K., Burden S.J., Chernoff J., Krauss R.S. Group I Paks promote skeletal myoblast differentiation *in vivo* and *in vitro*. Mol. Cell. Biol. 2017; 37:e00222-16. 10.1128/MCB.00222-16 PMC5288579 27920252

[50] Robin J.D. , Ludlow A.T., Batten K., Gaillard M.-C., Stadler G., Magdinier F., Wright W.E., Shay J.W. SORBS2 transcription is activated by telomere position effect-over long distance upon telomere shortening in muscle cells from patients with facioscapulohumeral dystrophy. Genome Res. 2015; 25:1781–1790. 26359233 10.1101/gr.190660.115 PMC4665000

[51] Hijazi M. , Smith R., Rajeeve V., Bessant C., Cutillas P.R. Reconstructing kinase network topologies from phosphoproteomics data reveals cancer-associated rewiring. Nat. Biotechnol. 2020; 38:493–502. 31959955 10.1038/s41587-019-0391-9

[52] Elkan C. The foundations of cost-sensitive learning. 2001; 2:973–978.

[53] Zhou Z.-H. , Liu X.-Y. Training cost-sensitive neural networks with methods addressing the class imbalance problem. IEEE Trans. Knowl. Data Eng. 2006; 18:63–77.

[54] Yang P. , Ormerod J.T., Liu W., Ma C., Zomaya A.Y., Yang J.Y.H. AdaSampling for positive-unlabeled and label noise learning with bioinformatics applications. IEEE Trans. Cybern. 2019; 49:1932–1943. 29993676 10.1109/TCYB.2018.2816984

[55] Diella F. , Cameron S., Gemünd C., Linding R., Via A., Kuster B., Sicheritz-Pontén T., Blom N., Gibson T.J. Phospho.ELM: a database of experimentally verified phosphorylation sites in eukaryotic proteins. BMC Bioinformatics. 2004; 5:79. 15212693 10.1186/1471-2105-5-79 PMC449700

[56] Yang C.-Y. , Chang C.-H., Yu Y.-L., Lin T.-C.E., Lee S.-A., Yen C.-C., Yang J.-M., Lai J.-M., Hong Y.-R., Tseng T.-L. et al. PhosphoPOINT: a comprehensive human kinase interactome and phospho-protein database. Bioinformatics. 2008; 24:i14–i20. 18689816 10.1093/bioinformatics/btn297

[57] Johnson J.L. , Yaron T.M., Huntsman E.M., Kerelsky A., Song J., Regev A., Lin T.-Y., Liberatore K., Cizin D.M., Cohen B.M. et al. An atlas of substrate specificities for the human serine/threonine kinome. Nature. 2023; 613:759–766. 36631611 10.1038/s41586-022-05575-3 PMC9876800

