# Supplementary material for: SnapKin: a snapshot deep learning ensemble for kinase-substrate prediction from phosphoproteomics data
Source: NAR Genom Bioinform. 2023 Nov 6;5(4):lqad099. doi: 10.1093/nargab/lqad099 (PMC10632189; doi:10.1093/nargab/lqad099)
Supplement: lqad099_Supplemental_Files [file lqad099_supplemental_files.zip › Supplementary table S1-S3 legends.docx]

Supplementary Table S1. Numeric results for creating Figure 2

Supplementary Table S2. Numeric results for creating Figure 3a

Supplementary Table S3. Numeric results for creating Figure 4a
